# Risk of developing open-angle glaucoma in patients with carotid artery stenosis: A nationwide cohort study

**DOI:** 10.1371/journal.pone.0194533

**Published:** 2018-04-23

**Authors:** Chien-Chih Chou, Min-Yen Hsu, Ching-Heng Lin, Che-Chen Lin, Chun-Yuan Wang, Ying-Cheng Shen, I-Jong Wang

**Affiliations:** 1 Department of Ophthalmology, Taichung Veterans General Hospital, Taichung, Taiwan; 2 Institute of Clinical Medicine, College of Medicine, National Taiwan University, Taipei, Taiwan; 3 Department of Ophthalmology, Chung Shan Medical University Hospital, Taichung, Taiwan; 4 Department of Medical Research, Taichung Veterans General Hospital, Taichung, Taiwan; 5 Healthcare Service Research Center (HSRC), Taichung Veterans General Hospital, Taichung, Taiwan; 6 Department of Ophthalmology, National Taiwan University Hospital, Taipei, Taiwan; National Eye Institute, UNITED STATES

## Abstract

Whether carotid artery stenosis (CAS) is an independent risk factor for open-angle glaucoma remains unclear. In this study, we investigated the association between CAS and the development of open-angle glaucoma in the Taiwanese population-based cohort derived from a longitudinal database containing claims data from the Taiwan National Health Insurance (NHI) program; this study enrolled 2093 patients with CAS and 8372 patients without CAS matched by age and sex from 1999 to 2010. Diagnoses of open-angle glaucoma (OAG) were identified during a follow-up period lasting through December 31, 2013. A Cox proportional hazards model was applied to evaluate the hazard ratio (HR) for OAG in the CAS cohort compared with the matched cohort. We found that the HR for open-angle glaucoma in the CAS cohort compared with the matched cohort. The adjusted HR for OAG in the CAS cohort was 1.50 (95% confidence interval, 1.11–2.02, *P* = .008). The Kaplan-Meier analysis revealed that the CAS cohort had a higher cumulative incidence of OAG than did the matched cohort during the follow-up period (log-rank test, *P* < .001). We concluded that CAS is a significantly independent risk factor for the development of OAG. Our finding is clinically important for the aging population, which has an increasing prevalence of CAS.

## Introduction

Ocular blood flow has been known as a major risk factor for glaucoma development and progression [1]. Although this association is difficult to study because different techniques are used for measuring different aspects of ocular circulation [2], reduced ocular blood flow has been known to occur in both early and late stages of glaucoma [3, 4]. This phenomenon can be observed within the optic nerve head [5], retinal circulation, choroid [6], retrobulbar [7], and even peripheral blood flow [8]. Reduced ocular blood flow seems to be more frequently found and more severe in normal-tension glaucoma than in high-tension glaucoma [9]. Furthermore, differences in blood flow reduction were more pronounced under provocation in glaucoma [10] and were also more pronounced in progressive than in nonprogressive eyes [11, 12]. Many studies have shown that the reduction of ocular blood flow definitely plays an important role in glaucoma development or progression [1, 13–22].

Reduced ocular blood flow has been known to be related to systemic hemodynamic changes. For example, the 9-year follow-up Barbados Eye Studies revealed that patients with lower systolic, diastolic, and mean ocular perfusion pressure levels had a significantly increased risk of developing open-angle glaucoma (OAG) (relative risk, 2.6 for low mean perfusion pressure [< 40 mmHg]). This longitudinal result supports the notion of reduced ocular perfusion pressure as a risk factor for OAG development [23]. In the Los Angeles Latino Eye Study, the authors found that lower diastolic (odds ratio [OR] = 1.9), systolic (OR = 2.5), and mean (OR = 3.6) ocular perfusion pressure levels were associated with a higher prevalence of OAG [24].

Patients with severe carotid artery diseases (CAS) may show retrobulbar hemodynamic changes and a greater risk of developing ocular ischemic syndrome [25]. Recently, Hayreh and Zimmerman enrolled 614 patients (728 eyes) with an ocular arterial occlusive disorder such as nonarteritic anterior ischemic optic neuropathy, central retinal artery occlusion, branch retinal artery occlusion, ocular ischemic syndrome, and amaurosis fugax [26]. They revealed that all patients had carotid artery diseases, and concluded that the ophthalmic artery originating from an atherosclerotic internal carotid artery has a markedly stenosed lumen at its origin. Under such conditions, ophthalmic artery stenosis is the primary cause of the reduction of ocular blood perfusion [26, 27]. However, they did not include patients with glaucoma in their cohort [26].

The role of carotid artery diseases in the pathogenesis of OAG has remained debatable in recent decades [1, 9, 28, 29]. In 1966, Moskovchenko et al analyzed 123 patients with OAG and concluded that the progression of the glaucomatous process was connected to sclerotic changes in the internal carotid and ophthalmic arteries in a number of patients [30]. However, Jampol et al followed 5 patients with severe bilateral CAS with increasing intraocular pressure for 3 to 12 years without developing glaucomatous disc or field changes [31]. Moreover, Bunin et al did not reveal any primary OAG in patients with CAS [32]. Greenfield and his colleagues also showed that the prevalence of hemodynamically significant CAS was not correlated with glaucoma severity [33].

Although most previous studies have suggested a correlation between reduced blood flow in various ocular tissues and OAG, whether CAS is a risk factor for OAG remains unclear. We hypothesized that the reduction of ocular perfusion caused by CAS would increase the risk of OAG. The objective of the current study was to investigate the association between CAS and the development of OAG in the Taiwanese population.

## Methods

### Study design and dataset

This was a population-based retrospective cohort study utilizing the Longitudinal Health Insurance Database (LHID2000), which is managed the Taiwan National Health Research Institute (NHRI). The Taiwan NHI program was introduced in 1995 and is mandatory for all citizens in Taiwan to join, except for people who are not regular residents of Taiwan. The coverage rate of this program is approximately 99% [34]. The LHID includes the claims data of 1 million randomly sampled individuals from the total 23 million NHI beneficiaries. The NHRI report revealed no statistically significant differences in age, sex, or area distribution between the cohorts in the LHID and in the Taiwan NHI [34]. This study was approved by the Ethics Review Board of Taichung Veterans General Hospital (CE13152B-3).

### Case definition

**Fig 1:**
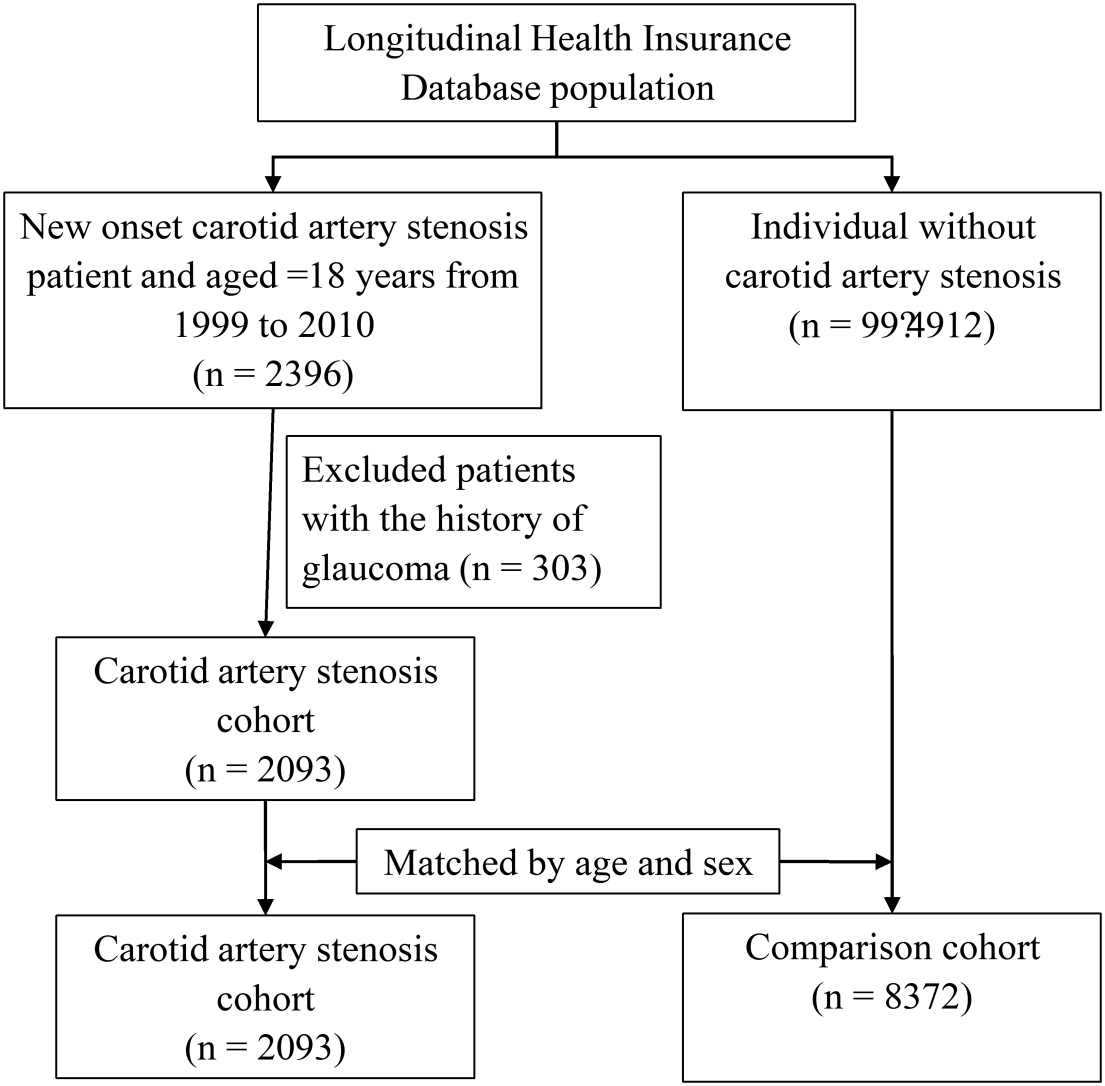
Flowchart of study population selection.

We enrolled patients with CAS aged ≥18 years as the study cohort. These patients had claims with the International Classification of Diseases, Ninth Revision, Clinical Modification (ICD-9-CM) diagnosis codes 433.10 and 433.11 from 1999 to 2010 from 1 inpatient or 3 outpatient visits. The index date was the date of an initial diagnosis of CAS. For the comparison group, we randomly selected patients from the LHID who did not receive a diagnosis of CAS and matched them to the patients by age and sex in a 4:1 ratio. The index date for patients in the comparison group was matched to the same date of the diagnosis of CAS in the case group. Patients with glaucoma (ICD-9-CM 365) before the index date in CAS patients and comparison subjects were excluded from the study. Fig 1 shows the flowchart for study population selection.

### Study outcomes

The primary outcome was diagnosis of incident open-angle glaucoma. For this study, the definition of open-angle glaucoma required the diagnosis of glaucoma or ocular hypertension (International Classification of Diseases, Ninth Revision, Clinical Modification, ICD-9: 365.X or 364.22). We excluded glaucoma patients with ICD-9: 365.2X, 364.22, 365.3X, 365.4X, 365.5X, and 365.6X or a history of glaucoma laser iridotomy or peripheral iridectomy for more strict definition for other ill-defined glaucoma in Taiwanese patients [35]. All of the patients had the same diagnosis at least 3 consequent visits and received the visual field examination during the visits. The censor of the study was defined as the subjects withdrawing the health insurance, glaucoma with laser iridotomy procedure or until December 31, 2013. Because several underlying diseases may affect open-angle glaucoma, we considered age, sex, hypertension (ICD-9-CM 401–405), hyperlipidemia (ICD-9-CM 272), diabetes (ICD-9-CM 250), migraine (ICD-9-CM 346), thyroid disease (ICD-9-CM 240–246), and heart failure (ICD-9-CM 428) as potential comorbidities in our study.[36] Carotid endarterectomy and carotid artery stenting are the surgical treatment options for CAS [37]. The number of topical antiglaucoma medications each patient was given was also analyzed. Combined antiglaucoma medications were considered to be 2 different antiglaucoma medications in our analysis.

### Statistical analysis

To examine the differences between the case cohort and the comparison cohort, a chi-squared test was used for categorical variables such as sex and comorbidities and the *t* test was applied for continuous variables. The hazard ratios (HRs) with 95% CIs of incident OAG were determined by the Cox proportional hazard model. The model was adjusted for age, sex, hypertension, hyperlipidemia, diabetes, migraine, TD and heart failure. The curve of cumulative incidence of OAG was assessed by the Kaplan-Meier method, with statistical significance examined by the log-rank test.

All analyses were performed using SAS version 9.4 (SAS Institute, Cary, NC, USA), and the incidence curve was plotted by R software (R Foundation for Statistical Computing, Vienna, Austria). All statistical tests were 2 sided, conducted at a significance level of 0.05, and reported using *P* values and 95% CIs.

## Results

Table 1 shows the demographic characteristics of the 2093 patients in the CAS cohort and the 8372 patients in the comparison cohort. Both cohorts exhibited similar distributions regarding sex and age. Both cohorts were predominantly composed of men (64.5%) and had an average age of 69 years. Compared with the comparison cohort, the CAS cohort had a significantly higher proportion of several comorbidities including hypertension (82.4% vs 57.6%), hyperlipidemia (47.1% vs 30.6%), diabetes (37.9% vs 24.5%), migraine (4.30% vs 2.44%), and heart failure (13.6% vs 8.09%).

**Table 1:** Demographic characteristics of patients with CAS (CAS cohort) and patients without CAS (comparison cohort).

Variable	Comparison cohort N = 8372	CAS cohort N = 2093	*P* value
Age, years (SD)	69.1 (11.8)	69.2 (11.7)	0.61
Sex			>0.99
Female	2972 (35.5)	743 (35.5)	
Male	5400 (64.5)	1350 (64.5)	
Comorbidity			
Hypertension	4821 (57.6)	1725 (82.4)	<0.0001
Hyperlipidemia	2558 (30.6)	986 (47.1)	<0.0001
Diabetes	2047 (24.5)	793 (37.9)	<0.0001
Migraine	204 (2.44)	90 (4.30)	<0.0001
TD	268 (3.20)	84 (4.01)	0.07
Heart failure	677 (8.09)	285 (13.6)	<0.0001

CAS, carotid artery stenosis; TD, thyroid disease.

**Fig 2:**
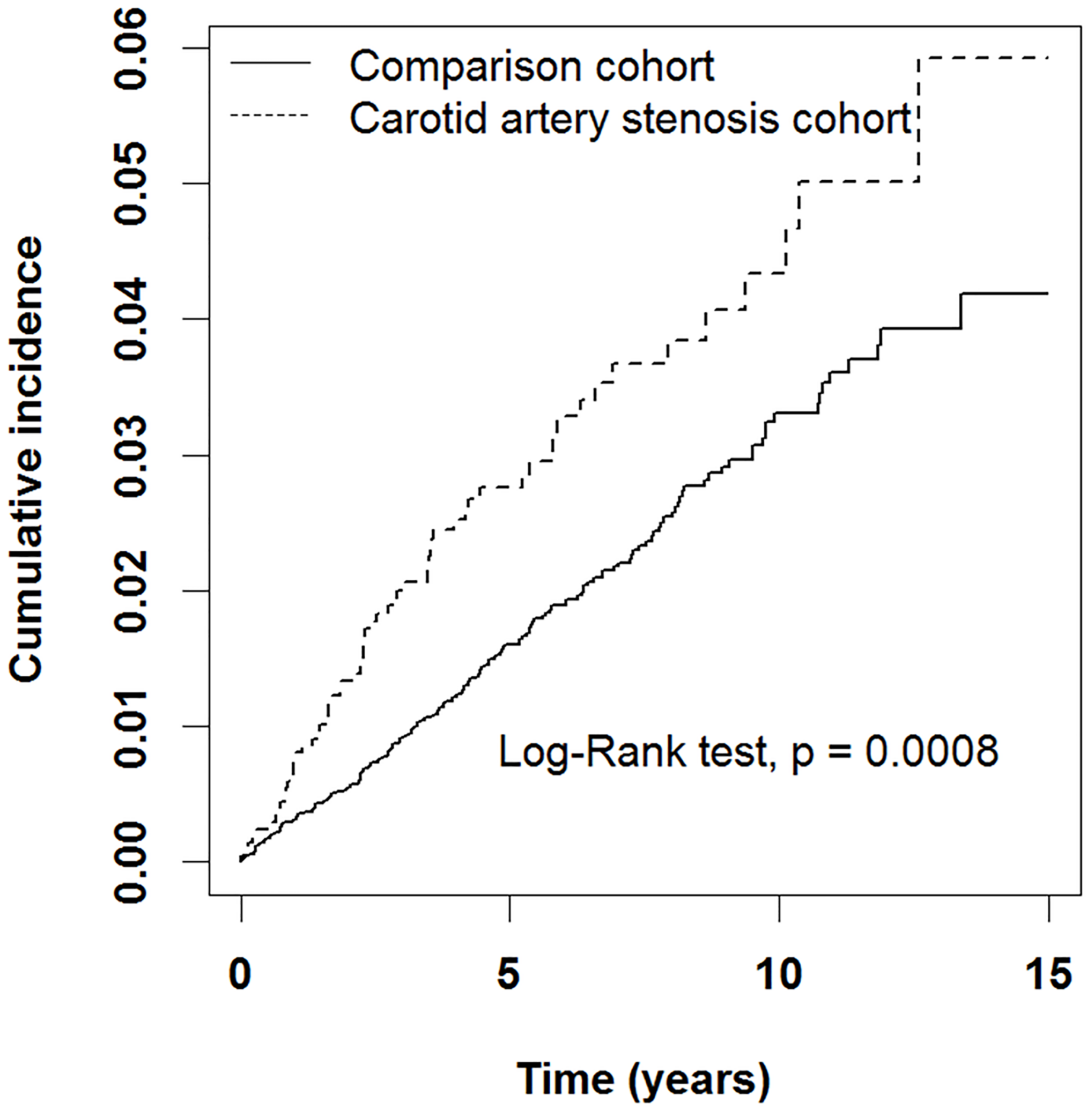
Cumulative incidence of open-angle glaucoma in patients with and without carotid artery stenosis. The carotid artery stenosis cohort had a higher cumulative incidence of open-angle glaucoma than did the matched cohort (*P <* .001 by log-rank test).

After adjustment for age, sex, hypertension, hyperlipidemia, diabetes, migraine, thyroid disease, and heart failure, patients with CAS still had a 1.50-fold higher risk of OAG than did the patients in the comparison group in our Cox model (Table 2; adjusted HR = 1.50, 95% CI, 1.11–2.02, *P* = .008). As illustrated in Fig 2, Kaplan-Meier analysis revealed that patients with CAS had a higher cumulative incidence of OAG than did the comparison cohort during the follow-up period (log-rank test, *P* = .0008). The median follow-up time and interquartile range for the CAS and comparison cohorts were 5.80 (5.03) and 5.12 (4.66) years, respectively.

**Table 2:** Incidence rate and HR of open-angle glaucoma in patients with carotid artery stenosis and the comparison cohort.

Outcomes	Event	Person-years	Incidence*	Crude HR (95% CI)	Adjusted HR** (95% CI)	p value
CAS						
No	174	54545	31.9	ref	ref	-
Yes	63	12079	52.2	1.63(1.22–2.17)	1.50(1.11–2.02)	0.008
Age						
<65	77	23019	33.5	ref	ref	-
65–75	109	25583	42.6	1.27(0.95–1.70)	1.19(0.88–1.61)	0.25
>75	51	18022	28.3	0.83(0.58–1.18)	0.76(0.53–1.10)	0.15
Sex						
Female	86	24527	35.1	ref	ref	-
Male	151	42096	35.9	1.02(0.78–1.33)	1.02(0.78–1.33)	0.91
Hypertension						
No	80	26834	29.8	ref	ref	-
Yes	157	39789	39.5	1.32(1.00–1.72)	1.14(0.84–1.55)	0.39
Hyperlipidemia						
No	149	45427	32.8	ref	ref	-
Yes	88	21196	41.5	1.26(0.96–1.63)	1.04(0.78–1.39)	0.78
Diabetes						
No	164	50357	32.6	ref	ref	-
Yes	73	16267	44.9	1.37(1.04–1.80)	1.22(0.90–1.64)	0.20
Migraine						
No	228	64995	35.1	ref	ref	-
Yes	9	1628	55.3	1.56(0.80–3.03)	1.48(0.76–2.89)	0.25
TD						
No	234	64534	36.3	ref	ref	-
Yes	3	2089	14.4	0.39(0.13–1.23)	0.36(0.11–1.13)	0.08
Heart failure						
No	216	62139	34.8	ref	ref	-
Yes	21	4485	46.8	1.33(0.85–2.08)	1.25(0.79–1.99)	0.34

*Incidence rate: per 10 000 person-years.

**Adjusted for age, sex, hypertension, hyperlipidemia, diabetes, migraine, TD and heart failure.

CAS, carotid artery stenosis; TD, thyroid disease; HR, hazard ratio.

After stratification by age and sex, comparing the risk of OAG in the CAS cohort with that in the comparison cohort showed that in the <65-year age group, patients with CAS were significantly associated with a 2.47-fold higher risk of developing OAG (Table 3; HR = 2.47, 95% CI, 1.49–4.09). Moreover, male patients with CAS had a 1.59-fold higher risk of OAG did those in the comparison group (Table 3; HR = 1.59, 95% CI, 1.09–2.31).

**Table 3:** Subgroup analysis stratified by age and sex.

Variables	Comparison cohort			CAS cohort			Adjusted HR (95% CI)	P value
	Event	PYs	Incidence*	Event	PYs	Incidence		
Age groups**								
<65	48	18851	25.5	29	4168	69.6	2.47(1.49–4.09)	0.0005
65–75	86	20928	41.1	23	4655	49.4	1.13(0.71–1.82)	0.60
>75	40	14766	27.1	11	3256	33.8	1.12(0.57–2.21)	0.74
Sex***								
Female	63	20067	31.4	23	4460	51.6	1.36(0.83–2.23)	0.22
Male	111	34478	32.2	40	7618	52.5	1.59(1.09–2.31)	0.02

*Incidence rate: per 10,000 person-years.

**adjusted for sex, hypertension, hyperlipidemia, diabetes, migraine, TD and heart failure.

***adjusted for age, hypertension, hyperlipidemia, diabetes, migraine, TD and heart failure.

PYs, patient-years; CAS, carotid artery stenosis; TD, thyroid disease.

The risk of developing OAG in the subgroup of CAS without surgical treatment was significantly higher than that in the comparison group (Table 4; HR = 1.57, 95% CI, 1.16–2.13). However, the risk of developing OAG in the subgroup of CAS with surgical treatment was not significantly different from that in the comparison group (HR = 0.79, 95% CI, 0.25–2.49).

**Table 4:** Risk of developing open-angle glaucoma in the comparison cohort, CAS without treatment subgroup, and CAS with treatment subgroup.

Variables	N	Event	PYs	Incidence	Adjusted HR (95% CI)	P value
Comparison cohort	8372	174	54545	31.9	ref	
CAS without treatment	1918	60	11043	54.3	1.57(1.16–2.13)	0.004
CAS with treatment	175	3	1036	29.0	0.79(0.25–2.49)	0.69

Incidence rate: per 10 000 person-years.

Treatment: surgical treatment including carotid endarterectomy and carotid artery stenting.

Adjusted for age, sex, hypertension, hyperlipidemia, diabetes, migraine, TD and heart failure.

PYs, patient-years; CAS, carotid artery stenosis; TD, thyroid disease.

In the comparison cohort, 39.1%, 48.3%, and 12.6% of patients with OAG received 0, 1, and 2 or more antiglaucoma medications prescribed at diagnosis, respectively. In the CAS cohort, 47.6%, 46.0%, and 6.35% of patients with OAG received 0, 1, and 2 or more antiglaucoma medications prescribed at diagnosis, respectively. Furthermore, in the comparison cohort, 27.4%, 38.2%, and 34.4% of patients with OAG received 0, 1, and 2 or more antiglaucoma medications prescribed 1 year after the diagnosis of OAG, respectively. In the CAS cohort, 34.5%, 37.9%, and 27.6% of patients with OAG received 0, 1, and 2 or more antiglaucoma medications prescribed 1 year after the diagnosis of OAG, respectively. However, there was no statistical difference in the number of antiglaucoma medications between both cohorts (Table 5).

**Table 5:** Number of antiglaucoma medications prescribed in patients with CAS and the comparison cohort.

Number of Antiglaucoma medications	At glaucoma diagnosis			1 year after the diagnosis of OAG		
	Comparison N = 174 (%)	CAS N = 63 (%)	*P* value	Comparison N = 157 (%)	CAS N = 58 (%)	*P* value
0	68 (39.1)	30 (47.6)	0.28	43 (27.4)	20 (34.5)	0.51
1	84 (48.3)	29 (46.0)		60 (38.2)	22 (37.9)	
2+	22 (12.6)	4 (6.35)		54 (34.4)	16 (27.6)	

CAS, carotid artery stenosis; OAG, open-angle glaucoma.

## Discussion

According to our results, Kaplan-Meier analysis revealed that patients with CAS had a higher cumulative incidence of OAG than did the comparison cohort during the follow-up period (log-rank test, *P* = .0008). Age, sex, hypertension, hyperlipidemia, diabetes mellitus, migraine, thyroid disease, and heart failure are known to be the risk factors for OAG [38]. After adjustment for these risk factors in our analysis, patients with CAS had a higher risk of OAG (adjusted HR = 1.50, 95% CI, 1.11–2.02). These results support our hypothesis that CAS is an independent risk factor for OAG. Our results are consistent with those of a previous study that showed a markedly higher rate of CAS in patients with OAG [39].

CAS results in reduced blood pressure in the ocular vascular bed, consequently engendering reduced ocular blood flow [26]. The results of the Los Angeles Latino Eye Study, Barbados Studies, Rotterdam Study, and studies in Hispanic adults support the notion of low ocular perfusion pressure as a risk factor for OAG development [23, 24, 40, 41]. Previous studies have also suggested reduced blood flow in various ocular tissues in patients with OAG [1, 42]. Cherecheanu et al proposed a model including primary and secondary insults in glaucoma. In this model, ischemia at the optic nerve head causes primary insult to retinal ganglion cell axons, whereas the ocular perfusion pressure falling below the limit of autoregulation will result in the secondary insults [43]. This proposed model can explain the results in our study.

Our results also demonstrate that patients with CAS aged <65 years were significantly associated with a 2.47-fold higher risk of OAG compared with the comparison cohort (HR = 2.47, 95% CI, 1.49–4.09). Male patients had a 1.59-fold higher risk of OAG than did those in the comparison cohort (HR = 1.59, 95% CI, 1.09–2.31). These results indicate that CAS is an important risk factor for OAG, particularly in male patients aged younger than 65 years. This is a novel finding that should be recognized as an independent risk factor in patients with OAG who are aged younger than 65 years.

Carotid endarterectomy and carotid artery stenting are the surgical treatment options for CAS [37]. The results of the Asymptomatic Carotid Surgery Trial [44] and the Asymptomatic Carotid Atherosclerosis Study have shown decreased risks of ipsilateral strokes at 5 years after carotid endarterectomy compared with medical therapy [45]. The North American Symptomatic Carotid Endarterectomy Trial also revealed that carotid endarterectomy could significantly reduce the incidence of ipsilateral cerebral events in patients with internal carotid artery greater than 70% stenosis [46]. Carotid artery stenting is a proposed alternative to carotid endarterectomy for both symptomatic and asymptomatic high-risk surgical candidates [47]. The current study also revealed a reduced HR of OAG with carotid endarterectomy or carotid artery stenting versus medical therapy. Our results imply that carotid endarterectomy or carotid artery stenting might improve ocular hemodynamics in patients with CAS and further reduce the risk of OAG. Our results are compatible with the findings of Kozobolis et al, which revealed that carotid endarterectomy could improve retrobulbar blood flow and perimetric parameters in patients with glaucoma,[48] and of other studies that have shown that carotid artery stenting could also improve chronic ocular ischemic syndrome and increase ocular circulation caused by severe CAS [49–51].

This study has the following limitations. First, clinical data such as intraocular pressure, central corneal thickness, visual field, optical coherence tomography, and optic nerve images are unavailable in the claims database. Second, CAS may have been underdiagnosed in this population because many patients with CAS are asymptomatic. Third, abnormal structure of the anterior chamber angle, genetic background, and CAS severity were also unavailable in our database. Fourth, OAG may have been underdiagnosed in this population because early-stage glaucoma has few warning signs or symptoms. Additional studies are required to confirm our epidemiological survey in order to clarify the underlying pathophysiological mechanisms responsible for the association between CAS and OAG.

## Conclusion

This is the first nationwide population-based study assessing the association between CAS and OAG. We found that patients with CAS had a significantly higher risk of OAG after adjustment for age, sex, and other confounders. This result indicates that CAS is an independent risk factor for OAG. Our finding is clinically important due to the increasing prevalence of CAS in the aging population. To facilitate early diagnosis and treatment, we suggest early referral of patients with CAS to ophthalmologists.

## References

[1] Flammer J, Orgul S, Costa VP, Orzalesi N, Krieglstein GK, Serra LM, et al The impact of ocular blood flow in glaucoma. Progress in retinal and eye research. 2002;21(4):359–93. Epub 2002/08/02. 12150988 10.1016/s1350-9462(02)00008-3

[2] Carter CJ, Brooks DE, Doyle DL, Drance SM. Investigations into a vascular etiology for low-tension glaucoma. Ophthalmology. 1990;97(1):49–55. 2314843 10.1016/s0161-6420(90)32627-1

[3] Michelson G, Langhans MJ, Harazny J, Dichtl A. Visual field defect and perfusion of the juxtapapillary retina and the neuroretinal rim area in primary open-angle glaucoma. Graefes Arch Clin Exp Ophthalmol. 1998;236(2):80–5. 9498117 10.1007/s004170050046

[4] Michelson G, Groh MJ, Groh ME, Grundler A. Advanced primary open-angle glaucoma is associated with decreased ophthalmic artery blood-flow velocity. Ger J Ophthalmol. 1995;4(1):21–4. 7728106

[5] Harju M, Vesti E. Blood flow of the optic nerve head and peripapillary retina in exfoliation syndrome with unilateral glaucoma or ocular hypertension. Graefes Arch Clin Exp Ophthalmol. 2001;239(4):271–7. 11450491 10.1007/s004170100269

[6] Yin ZQ, Vaegan, Millar TJ, Beaumont P, Sarks S. Widespread choroidal insufficiency in primary open-angle glaucoma. J Glaucoma. 1997;6(1):23–32. 9075077

[7] Rankin SJ. Color Doppler imaging of the retrobulbar circulation in glaucoma. Survey of ophthalmology. 1999;43 Suppl 1:S176–82. 10416761 10.1016/s0039-6257(99)00043-0

[8] Choi J, Kook MS. Systemic and Ocular Hemodynamic Risk Factors in Glaucoma. Biomed Res Int. 2015;2015:141905 doi: 10.1155/2015/141905 26557650 10.1155/2015/141905 PMC4628774

[9] Drance S. Chronic open angle glaucoma: risk factors in addition to intraocular pressure. Acta ophthalmologica Scandinavica. 2001;79(6):545 Epub 2002/01/10. 11782217 10.1034/j.1600-0420.2001.790601.x

[10] Flammer J. The vascular concept of glaucoma. Survey of ophthalmology. 1994;38 Suppl:S3–6. 7940146 10.1016/0039-6257(94)90041-8

[11] Yamazaki Y, Drance SM. The relationship between progression of visual field defects and retrobulbar circulation in patients with glaucoma. American journal of ophthalmology. 1997;124(3):287–95. 9439354 10.1016/s0002-9394(14)70820-7

[12] Stewart WC, Kolker AE, Sharpe ED, Day DG, Holmes KT, Leech JN, et al Factors associated with long-term progression or stability in primary open-angle glaucoma. American journal of ophthalmology. 2000;130(3):274–9. 11020404 10.1016/s0002-9394(00)00487-6

[13] Flammer J, Orgül S. Optic nerve blood-flow abnormalities in glaucoma. Progress in retinal and eye research. 1998;17(2):267–89. 9695795 10.1016/s1350-9462(97)00006-2

[14] Kaiser HJ, Schoetzau A, STÜMPFIG D, Flammer J. Blood-flow velocities of the extraocular vessels in patients with high-tension and normal-tension primary open-angle glaucoma. American journal of ophthalmology. 1997;123(3):320–7. 9063241 10.1016/s0002-9394(14)70127-8

[15] Harris A, Sergott R, Spaeth G, Katz J, Shoemaker J, Martin B. Color Doppler analysis of ocular vessel blood velocity in normal-tension glaucoma. American journal of ophthalmology. 1994;118(5):642–9. 7977577 10.1016/s0002-9394(14)76579-1

[16] Nicolela MT, Hnik P, Drance SM. Scanning laser Doppler flowmeter study of retinal and optic disk blood flow in glaucomatous patients. American journal of ophthalmology. 1996;122(6):775–83. 8956631 10.1016/s0002-9394(14)70373-3

[17] Sugiyama T, Araie M, Riva CE, Schmetterer L, Orgul S. Use of laser speckle flowgraphy in ocular blood flow research. Acta ophthalmologica. 2010;88(7):723–9. doi: 10.1111/j.1755-3768.2009.01586.x 19725814 10.1111/j.1755-3768.2009.01586.x

[18] Kobayashi W, Kunikata H, Omodaka K, Togashi K, Ryu M, Akiba M, et al Correlation of optic nerve microcirculation with papillomacular bundle structure in treatment naive normal tension glaucoma. Journal of ophthalmology. 2014;2014. 10.1155/2014/468908 PMC4276121 25574382

[19] Aizawa N, Kunikata H, Shiga Y, Yokoyama Y, Omodaka K, Nakazawa T. Correlation between structure/function and optic disc microcirculation in myopic glaucoma, measured with laser speckle flowgraphy. BMC ophthalmology. 2014;14(1):113. 25252729 10.1186/1471-2415-14-113 PMC4194365

[20] Shiga Y, Omodaka K, Kunikata H, Ryu M, Yokoyama Y, Tsuda S, et al Waveform Analysis of Ocular Blood Flow and the Early Detection of Normal Tension GlaucomaWaveform Changes in Ocular Blood Flow. Investigative ophthalmology & visual science. 2013;54(12):7699–706. 24130177 10.1167/iovs.13-12930

[21] Aizawa N, Kunikata H, Yokoyama Y, Nakazawa T. Correlation between optic disc microcirculation in glaucoma measured with laser speckle flowgraphy and fluorescein angiography, and the correlation with mean deviation. Clinical & experimental ophthalmology. 2014;42(3):293–4. 23601712 10.1111/ceo.12130

[22] Nakazawa T. Ocular blood flow and influencing factors for glaucoma. The Asia-Pacific Journal of Ophthalmology. 2016;5(1):38–44. doi: 10.1097/APO.0000000000000183 26886118 10.1097/APO.0000000000000183

[23] Leske MC, Wu SY, Hennis A, Honkanen R, Nemesure B. Risk factors for incident open-angle glaucoma: the Barbados Eye Studies. Ophthalmology. 2008;115(1):85–93. Epub 2007/07/17. doi: 10.1016/j.ophtha.2007.03.017 17629563 10.1016/j.ophtha.2007.03.017

[24] Memarzadeh F, Ying-Lai M, Chung J, Azen SP, Varma R. Blood pressure, perfusion pressure, and open-angle glaucoma: the Los Angeles Latino Eye Study. Investigative ophthalmology & visual science. 2010;51(6):2872–7. 20089880 10.1167/iovs.08-2956 PMC2891455

[25] Costa VP, Kuzniec S, Molnar LJ, Cerri GG, Puech-Leao P, Carvalho CA. Clinical findings and hemodynamic changes associated with severe occlusive carotid artery disease. Ophthalmology. 1997;104(12):1994–2002. Epub 1997/12/24. 9400757 10.1016/s0161-6420(97)30066-9

[26] Hayreh SS, Zimmerman MB. Ocular arterial occlusive disorders and carotid artery disease. Ophthalmology Retina. 2017;1(1):12–8. doi: 10.1016/j.oret.2016.08.003 28547004 10.1016/j.oret.2016.08.003 PMC5439962

[27] Hayreh SS, Dass R. THE OPHTHALMIC ARTERY: I. ORIGIN AND INTRA-CRANIAL AND INTRA-CANALICULAR COURSE. The British journal of ophthalmology. 1962;46(2):65–98. Epub 1962/02/01. 18170762 10.1136/bjo.46.2.65 PMC510170

[28] Anderson DR. Introductory comments on blood flow autoregulation in the optic nerve head and vascular risk factors in glaucoma. Survey of ophthalmology. 1999;43 Suppl 1:S5–9. Epub 1999/07/23. 10416742 10.1016/s0039-6257(99)00046-6

[29] Huck A, Harris A, Siesky B, Kim N, Muchnik M, Kanakamedala P, et al Vascular considerations in glaucoma patients of African and European descent. Acta ophthalmologica. 2014;92(5):e336–40. Epub 2014/01/28. doi: 10.1111/aos.12354 24460758 10.1111/aos.12354 PMC4107083

[30] Moskovchenko KP. [On the development of glaucoma in patients with disturbed blood circulation in the carotid artery system]. Vestn Oftalmol. 1966;79(6):48–51. 5994162

[31] Jampol LM, Miller NR. Carotid artery disease and glaucoma. The British journal of ophthalmology. 1978;62(5):324–6. 656358 10.1136/bjo.62.5.324 PMC1043222

[32] Bunin A, Mukha AI, Kolomoitseva EM. [Perfusion pressure in ocular vessels of patients with open-angle glaucoma]. Vestn Oftalmol. 1995;111(1):28–31. 7771039

[33] Greenfield DS, Bagga H. Blood flow studies and serological testing in the diagnostic evaluation of glaucoma: a pilot study. Ophthalmic Surg Lasers Imaging. 2004;35(5):406–14. 15497551

[34] Wu T-Y, Majeed A, Kuo KN. An overview of the healthcare system in Taiwan. London journal of primary care. 2010;3(2):115–9. 25949636 10.1080/17571472.2010.11493315 PMC3960712

[35] Hwang DK, Liu CJ, Pu CY, Chou YJ, Chou P. Persistence of topical glaucoma medication: a nationwide population-based cohort study in Taiwan. JAMA Ophthalmol. 2014;132(12):1446–52. doi: 10.1001/jamaophthalmol.2014.3333 25211300 10.1001/jamaophthalmol.2014.3333

[36] Chiang SJ, Daimon M, Wang LH, Hung MJ, Chang NC, Lin HC. Association between mitral valve prolapse and open-angle glaucoma. Heart (British Cardiac Society). 2015;101(8):609–15. Epub 2014/12/17. 25502813 10.1136/heartjnl-2014-306198

[37] Spacek M, Veselka J. Carotid artery stenting—current status of the procedure. Archives of medical science: AMS. 2013;9(6):1028–34. Epub 2014/02/01. doi: 10.5114/aoms.2013.39216 24482646 10.5114/aoms.2013.39216 PMC3902709

[38] Quigley HA. Open-angle glaucoma. The New England journal of medicine. 1993;328(15):1097–106. doi: 10.1056/NEJM199304153281507 8455668 10.1056/NEJM199304153281507

[39] Bulboaca A, Bulboaca A, Nicula C. [Endothelial dysfunction in patients with open-angle glaucoma and atheromatous lesions]. Oftalmologia (Bucharest, Romania: 1990). 2003;58(3):52–5. Epub 2004/01/02. 14702733

[40] Quigley HA, West SK, Rodriguez J, Munoz B, Klein R, Snyder R. The prevalence of glaucoma in a population-based study of Hispanic subjects: Proyecto VER. Archives of ophthalmology (Chicago, Ill 1960). 2001;119(12):1819–26. Epub 2001/12/26. 10.1001/archopht.119.12.1819 11735794

[41] Hulsman CA, Vingerling JR, Hofman A, Witteman JC, de Jong PT. Blood pressure, arterial stiffness, and open-angle glaucoma: the Rotterdam study. Archives of ophthalmology (Chicago, Ill: 1960). 2007;125(6):805–12. 10.1001/archopht.125.6.805 17562992

[42] Harris A, Kagemann L, Ehrlich R, Rospigliosi C, Moore D, Siesky B. Measuring and interpreting ocular blood flow and metabolism in glaucoma. Canadian journal of ophthalmology Journal canadien d’ophtalmologie. 2008;43(3):328–36. Epub 2008/04/30. doi: 10.3129/i08-051 18443609 10.3129/i08-051

[43] Cherecheanu AP, Garhofer G, Schmidl D, Werkmeister R, Schmetterer L. Ocular perfusion pressure and ocular blood flow in glaucoma. Current opinion in pharmacology. 2013;13(1):36–42. doi: 10.1016/j.coph.2012.09.003 23009741 10.1016/j.coph.2012.09.003 PMC3553552

[44] Halliday A, Mansfield A, Marro J, Peto C, Peto R, Potter J, et al Prevention of disabling and fatal strokes by successful carotid endarterectomy in patients without recent neurological symptoms: randomised controlled trial. Lancet (London, England). 2004;363(9420):1491–502. Epub 2004/05/12. 10.1016/S0140-6736(04)16146-1 15135594

[45] Endarterectomy for asymptomatic carotid artery stenosis. Executive Committee for the Asymptomatic Carotid Atherosclerosis Study. Jama. 1995;273(18):1421–8. Epub 1995/05/10. 7723155

[46] Barnett HJM, Taylor DW, Haynes RB, Sackett DL, Peerless SJ, Ferguson GG, et al Beneficial effect of carotid endarterectomy in symptomatic patients with high-grade carotid stenosis. The New England journal of medicine. 1991;325(7):445–53. Epub 1991/08/15. doi: 10.1056/NEJM199108153250701 1852179 10.1056/NEJM199108153250701

[47] Mughal MM, Khan MK, DeMarco JK, Majid A, Shamoun F, Abela GS. Symptomatic and asymptomatic carotid artery plaque. Expert review of cardiovascular therapy. 2011;9(10):1315–30. Epub 2011/10/12. doi: 10.1586/erc.11.120 21985544 10.1586/erc.11.120 PMC3243497

[48] Kozobolis VP, Detorakis ET, Georgiadis GS, Achtaropoulos AA, Papas TT, Lazarides MK. Perimetric and retrobulbar blood flow changes following carotid endarterectomy. Graefe’s archive for clinical and experimental ophthalmology = Albrecht von Graefes Archiv fur klinische und experimentelle Ophthalmologie. 2007;245(11):1639–45. Epub 2007/04/26. 10.1007/s00417-007-0589-2 17457602

[49] Arai N, Sasahara A, Hagiwara S, Tani S, Ohbuchi H, Hirota K, et al [Immediate improvement of ischemic oculopathy after stenting for internal carotid artery stenosis]. Brain and nerve = Shinkei kenkyu no shinpo. 2014;66(12):1503–8. Epub 2014/12/06. doi: 10.11477/mf.1416200065 25475037 10.11477/mf.1416200065

[50] Kawaguchi S, Sakaki T, Iwahashi H, Fujimoto K, Iida J-i, Mishima H, et al Effect of carotid artery stenting on ocular circulation and chronic ocular ischemic syndrome. Cerebrovascular Diseases. 2006;22(5–6):402–8. doi: 10.1159/000094859 16888383 10.1159/000094859

[51] Kawaguchi S, Iida J-i, Uchiyama Y. Ocular circulation and chronic ocular ischemic syndrome before and after carotid artery revascularization surgery. Journal of ophthalmology. 2012;2012. 10.1155/2012/350475 PMC3536051 23316337

